# Crimean-Congo Hemorrhagic Fever Virus, Greece

**DOI:** 10.3201/eid2002.130690

**Published:** 2014-02

**Authors:** Anna Papa, Persefoni Sidira, Victor Larichev, Ludmila Gavrilova, Ksenia Kuzmina, Mehrdad Mousavi-Jazi, Ali Mirazimi, Ute Ströher, Stuart Nichol

**Affiliations:** Aristotle University of Thessaloniki School of Medicine, Thessaloniki, Greece (A. Papa, P. Sidira);; D.I. Ivanovsky Institute of Virology, Moscow, Russian Federation (V. Larichev, L. Gavrilova, K. Kuzmina);; Swedish Institute for Communicable Disease Control, Stockholm, Sweden (M. Mousavi-Jazi, A. Mirazimi);; National Veterinary Institute, Uppsala, Sweden (A. Mirazimi);; Linköpings University Institute of Clinical and Experimental Medicine, Linköping, Sweden (A. Mirazimi);; Centers for Disease Control and Prevention, Atlanta, Georgia, USA (U. Ströher, S. Nichol)

**Keywords:** Crimean-Congo hemorrhagic fever virus, CCHFV, viruses, ELISA, immunofluorescence assay, neutralization test, Greece, vector-borne infections, ticks, seroprevalence

## Abstract

Seroprevalence of Crimean-Congo hemorrhagic fever virus (CCHFV) is high in some regions of Greece, but only 1 case of disease has been reported. We used 4 methods to test 118 serum samples that were positive for CCHFV IgG by commercial ELISA and confirmed the positive results. A nonpathogenic or low-pathogenicity strain may be circulating.

Crimean-Congo hemorrhagic fever (CCHF) virus (CCHFV; genus *Nairovirus*, family *Bunyaviridae*) causes severe disease in humans and circulates in many areas of Africa, Asia, and Europe. The disease is characterized by a sudden onset of high fever, chills, severe headache, dizziness, and back and abdominal pains; additional signs and symptoms include nausea, vomiting, diarrhea, and neuropsychiatric and cardiovascular changes. In severe cases, hemorrhagic manifestations are present, ranging from petechiae to large areas of ecchymosis and bleeding from different sites of the body. Case-fatality rates range from 10% to 50% (*1*). Humans become infected with CCHFV through tick bites, mainly from *Hyalomma* spp. ticks, or by direct contact with blood or tissues from viremic livestock or infected humans. The geographic range of CCHFV is the most extensive among tick-borne viruses related to human health, coinciding with the distribution of *Hyalomma* spp. ticks (*1*).

Greece is a country in the south of the Balkan Peninsula. Although sporadic cases or outbreaks of CCHF are often observed in other Balkan countries, including Bulgaria, Albania, and Kosovo (*2*–*5*), only 1, fatal, CCHF case has been reported in Greece (*6*). The causative strain, Rhodopi-2008, clusters with other pathogenic Balkan CCHFV strains; however, it differs by >20% at the nucleotide level from the CCHFV strain AP92, which was isolated from *Rhipicephalus bursa* ticks collected in 1975 from goats in Vergina village in northern Greece (*7*). Human disease has not been associated with the AP92 strain, and only a few mild cases associated with an AP92-like strain have been reported in Turkey (*8*). 

Recent studies have shown that the seroprevalence of CCHFV antibodies (IgG) in the general population of Greece is ≈4%, but large differences are seen between regions (range 0%–27%) (*9*–*11*). All of these studies used commercial ELISA kits to detect CCHFV IgG. Our aim was to retest these IgG-positive samples by using additional serologic methods to evaluate the accuracy of the results of the earlier studies.

## The Study

We investigated 118 serum samples that had shown positive test results for CCHFV IgG during 3 recent seroprevalence studies in Greece (*9*–*11*). The ages of the seropositive persons ranged from 26 to 90 years (median 73 years). Oral consent had been given by all participants, and the seroprevalence studies were approved by the Ethics Committee of the Medical School of Aristotle University of Thessaloniki, Greece. For control, 20 CCHFV IgG–negative samples were tested. All positive samples had been tested by using a commercial ELISA (Vektor-Best, Novosibirsk, Russia) and stored at −70°C.

We tested all the samples by 3 methods: 1) an in-house, sandwich-capture ELISA described by the Ivanovsky Institute of Virology (Moscow, Russia), which uses inactivated sucrose-acetone–extracted whole-cell antigen of CCHFV strain UZ10145 prepared from the brain of infected newborn white mice (*12*); 2) an in-house sandwich/indirect ELISA developed by the Centers for Disease Control and Prevention (Atlanta, GA, USA), which uses cells infected with CCHFV strain IbAr 10200 as the antigen (*13*); and 3) a commercial immunofluorescence assay (IFA; CCHFV Mosaic 2, Euroimmun Medizinische Labordiagnostika AG, Lübeck, Germany), which uses cells transfected with CCHFV glycoprotein and nucleoprotein (CCHFV strain IbAr 10200) and control-transfected cells. Serum dilution of 1:100 was used in both ELISAs. IFA was performed according to the manufacturer’s instructions by using 1:100 serum dilution; when a negative result was obtained, the test was repeated at lower dilutions (1:40 and 1:16). In addition to these methods, we tested 48 of the 118 CCHFV IgG-positive samples by microneutralization assay using the CCHFV strain IbAr 10200; the samples selected for this test method were from all geographic regions of Greece (*14*). This procedure was performed in a high-containment Biosafety Level 4 laboratory at the Karolinska Institute (Stockholm, Sweden).

ELISA results were noted as high-positive, positive, or low-positive according to the optical density in relation to the respective cutoff value. IFA grading was done on the basis of serum dilution: 1:16, low-positive; 1:40, positive; and >1:100, high-positive.

Both ELISAs and the IFA found CCHFV IgG in 116 (98.3%) of 118 samples (Table). One sample was negative by all methods, including the neutralization test, and 1 sample had positive results on 1 ELISA and showed a low-positive result by IFA (this sample was not tested by neutralization test). The microneutralization assay detected CCHFV in 47/48 samples tested; titers ranged from 10 to 200 (mean 105). All IgG-negative samples had negative results by both ELISAs and by IFA.

**Table:** Results of ELISA and IFA testing of 118 serum samples initially positive for CCHFV by ELISA, Greece*

Result	No. (%) samples			
	Initial ELISA†	CDC ELISA‡	Ivanovsky Institute ELISA§	IFA¶
High-positive	76 (64.4)	61 (51.7)	73 (61.8)	78 (66.1)
Positive	29 (24.6)	43 (36.4)	30 (25.4)	26 (22.0)
Low-positive	13 (11.0)	13 (11.0)	13 (11.0)	13 (11.0)
Negative	NA	1 (0.8)	2 (1.7)	1 (0.8)

*IFA, immunofluorescence assay; CCHFV, Crimean-Congo hemorrhagic fever virus; CDC, US Centers for Disease Control and Prevention; NA, not applicable. †Samples were initially collected and tested for IgG by ELISA (Vektor-Best, Novosibirsk, Russia) as part of 3 previous seroprevalence studies (*9**–**11*). ‡CDC, Atlanta, Georgia, USA (*13*). §Ivanovsky Institute of Virology, Moscow, Russia (*12*). ¶CCHFV Mosaic 2; Euroimmun Medizinische Labordiagnostika AG, Lübeck, Germany.

## Conclusions

Our results confirm previous findings that the CCHFV seroprevalence in Greece is high, especially in areas where livestock husbandry is a major occupation. All the methods used to test these samples produced similar results, which suggests that these methods are reliable laboratory tools for CCHFV seroprevalence studies. The commercial ELISA previously used had been used in various seroprevalence studies, including a study performed in response to a CCHF outbreak in western Afghanistan (*15*). That study reported (as unpublished data) that this kit had been previously compared with an in-house ELISA from the US Army Medical Research Institute for Infectious Diseases (Fort Detrick, MD, USA), and results of the commercial and in-house ELISAs were comparable for all samples tested.

An advantage of the in-house ELISAs, which also run each sample with a negative antigen (background), is that they efficiently reduce the number of false-positive results. The commercial IFA includes control-transfected cells for the same reason. Furthermore, this IFA enables the detection of antibodies against CCHFV nucleoprotein or glycoprotein antigens. The viral antigens in this specific IFA and in the applied in-house ELISAs are expressed in eukaryotic cells, which suggests that the glycosylation and 3D structure of the viral proteins were authentic in the assays we used.

Neutralization testing for CCHFV is a relatively difficult technique because the procedure must be performed in a Biosafety Level 4 environment and because nairoviruses produce weaker neutralizing antibody responses than do members of other *Bunyaviridae* family genera (*1*). In our study, CCHFV-neutralizing antibodies were detected in all but 1 of the samples tested.

Given that only 1 CCHF case has been reported in Greece, the high seroprevalence in specific regions might be related to a nonpathogenic or low-pathogenicity strain, such as the CCHFV strain AP92. The confirmation of the high CCHFV seroprevalence in specific areas of Greece needs further investigation to detect undiagnosed CCHF cases or elucidate the factors playing a role in subclinical infections.
